# A Study of BMP-2-Loaded Bipotential Electrolytic Complex around a Biphasic Calcium Phosphate-Derived (BCP) Scaffold for Repair of Large Segmental Bone Defect

**DOI:** 10.1371/journal.pone.0163708

**Published:** 2016-10-06

**Authors:** Kallyanashis Paul, Andrew R. Padalhin, Nguyen Thuy Ba Linh, Boram Kim, Swapan Kumar Sarkar, Byong Taek Lee

**Affiliations:** 1 Department of Regenerative Medicine, College of Medicine, Soonchunhyang University, 366–1 Ssangyong dong, Cheonan, 330–090, South Korea; 2 Institute of Tissue Regeneration, College of Medicine, Soonchunhyang University, 366–1 Ssangyong dong, Cheonan, 330–090, South Korea; University of Connecticut Health Center, UNITED STATES

## Abstract

A bipotential polyelectrolyte complex with biphasic calcium phosphate (BCP) powder dispersion provides an excellent option for protein adsorption and cell attachment and can facilitate enhanced bone regeneration. Application of the bipotential polyelectrolyte complex embedded in a spongy scaffold for faster healing of large segmental bone defects (LSBD) can be a promising endeavor in tissue engineering application. In the present study, a hollow scaffold suitable for segmental long bone replacement was fabricated by the sponge replica method applying the microwave sintering process. The fabricated scaffold was coated with calcium alginate at the shell surface, and genipin-crosslinked chitosan with biphasic calcium phosphate (BCP) dispersion was loaded at the central hollow core. The chitosan core was subsequently loaded with BMP-2. The electrolytic complex was characterized using SEM, porosity measurement, FTIR spectroscopy and BMP-2 release for 30 days. *In vitro* studies such as MTT, live/dead, cell proliferation and cell differentiation were performed. The scaffold was implanted into a 12 mm critical size defect of a rabbit radius. The efficacy of this complex is evaluated through an *in vivo* study, one and two month post implantation. BV/TV ratio for BMP-2 loaded sample was (42±1.76) higher compared with hollow BCP scaffold (32±0.225).

## Introduction

Recent advancements in bone tissue regeneration have considerable and worthwhile impact in the field of large segmental bone defects (LSBD), congenital deformities, and open fractures. A considerable challenge in orthopedics for large segmental bone defects repair arising from severe trauma, tumor resection, and tibia fractures, still remains. Currently used bone grafts such as vascularized autologous, heterologous (xenograft), and homologous bone grafts are associated with donor site morbidity, limited availability, possible immune response, possible animal derived pathogen transmission, and risk of immunogenic rejection [1, 2]. Synthetic bone graft is a promising means to overcome these problems.

Synthetic bone graft is gaining growing attention due to the availability of ever increasing suitable fabrication techniques, ability to mimic bone microstructure, and requisite mechanical properties that are adjustable to specific functional applications. The successful synthetic bone grafts are capable of osteoconductivity and osteoindictivity and to support angiogenesis in the formation of new bone growth [3]. In this regard, biphasic calcium phosphate powder (BCP, a combination of hydroxyappatite and tri calcium phosphate) derived spongy scaffolds with bioactive polymer modification has already been demonstrated as a promising synthetic bone grafts for hard tissue regeneration.

BCP spongy scaffold fabricated by a microwave assisted sintering process is reportedly an established osteoconductive bone graft with a 3D interconnected porous structure. It favors cell attachment, proliferation, differentiation, and also provides pathways for physiological fluids. In the present work, we fabricated hollow spongy scaffolds with optimum pore size distribution to mimic bone microstructure. The design strategy was to enable the scaffold to guide the regeneration process in such a way that it ultimately produces the long bone macroarchitecture conforming and integrating with the native living long bone of the patient.

Loading biopolymers around a BCP hollow spongy scaffold can enhance mechanical stability and act as a potential carrier for protein based drugs such as BMP-2, platelet derived growth factors [4–6]. In the present work, we loaded BCP dispersed chitosan as a natural cationic polymer at the core of the hollow spongy scaffold, and calcium alginate as an anionic polymer at the shell. Chitosan is crosslinked with genipin for increasing the stability in physiological condition. BCP loading might enhance hydrophilicity inside hydrophobic chitosan. This changeover is favorable for guided bone regeneration. In addition, positively charged proteins can bind to the negatively charged calcium alginate at the shell of the scaffold. Thus, this bio-polymer composition can function as a bipotential bio-active surface with enhanced cellular binding sites for cell adhesion, proliferation, and differentiation, and promote cellular migration inside the scaffold. As glycosaminoglycans (GAGs) or mucopolysaccharides such as heparin sulphate (HS) and chondroitin sulphate (CS) are negatively charged, they can act as binding sites to the positively charged chitosan at the core of the fabricated scaffold [7]. Polyelectrolytic (PEC) surfaces can bind protein based drugs and can act as efficient drug delivery vehicles. This bipotential polymeric arrangement, coupled with its favorable biodegradability, biocompatibility, and non-toxicity, can perform biochemically well in hemostasis and angiogenesis [8].

BMP-2 is one of the potential candidates to promote bone and cartilage inductive activity [9]. BMP-2 has critical roles in the initiation of the endochondral pathway to enhance bone formation during mammalian development [10]. In the present work, BMP-2 is adsorbed effectively into BCP powder loaded chitosan and thereby osteoinductivity is induced. Electrostatic attraction between HAP and BMP-2 is already reported [11]. This BMP-2-loaded bipotential surface favors the biological response such as cell attachment behavior, blood coagulation, and complete fusion of bio-fluids into the implantation site [12].

Thus, using this design it is possible to envisage a favorable environment in which to associate autologous cells and proteins that would promote cell adhesion with an osteoconductive material, in order to create osteoinductive materials in the healing zone [13]. The experiments performed herein reveal the degree of bone regeneration potential of a BMP-2-loaded bipotential osteoinductive hollow spongy scaffold.

## Materials and Methods

### 2.1 Materials

Medium-molecular-weight chitosan from shrimp shells (*M*w: 645kDa, ≥75%, deacetylated) and medium viscosity (≥2,000 Pa, 2% (25°C; lit.)) alginic acid sodium salt (extracted from brown algae) were purchased from Sigma-Aldrich, United Kingdom. Anhydrous calcium chloride (96.0%) for transforming calcium alginate was obtained from Samchun Pure Chemical Co, Ltd., Korea. BCP powder was synthesized through a microwave assisted hydrothermal process [14, 15] followed by calcination at 900°C for 1hr. High crystallinity is confirmed by XRD.

For the *in vitro* study, dimethylsulfoxide (99.0%) (DMSO) was purchased from Samchun Pure Chemical Co, Ltd., Korea. Fetal bovine serum (FBS), penicillin-streptomycin antibiotics (PS), 3-[4,5-dimethylthiazol-2-yl] 2,5 diphenyltetrazolium bromide (MTT) solution, and trypsin-EDTA were purchased from Gibco (Carlsbad, CA). Phosphate-buffered saline (PBS) tablets were obtained from Amresco, Korea. MC3T3-E1 mouse pre-osteoblast cells were obtained from the American Type Culture Collection (subclone 4, ATCC, USA). The MC3T3-E1 cells were maintained in a humidified incubator at 37°C and 5% CO_2_.New Zealand white rabbits (weight 3kg) were purchased commercially and treated according to the guidelines provided by the Soonchunhyang University.

### 2.2 Preparation of hollow BCP spongy scaffolds

The BCP scaffold was fabricated using the sponge replica method described in our previous study [16]. Briefly, the polyurethane sponge (60ppi, HD sponge) was immersed in BCP slurry containing 5% (w/v) Butvar and 25% (w/v) BCP powder. This immersed BCP scaffold was taken out from the solution and sprayed until the removal of excess slurry from the surface. This process transformed the spongy scaffold with uniform coating thickness. Total cycle (dipping and spraying) was repeated 5 times to obtain scaffold with desired strength. At the end of each cycle, samples were perfectly dried inside 80°C oven. After that the final scaffolds were sintered in a microwave furnace (UMF-01, 2.45GHz; Unicera) at 1200°C for 10 minutes, with a heating rate of 100°C/min. By this process, any remnants from polyurethane sponge were removed. The sponge BCP scaffolds were autoclaved to remove any contamination.

### 2.3 Bipotential polymer coating around the hollow BCP spongy scaffold

The negative potential polymer solution: sodium alginate was mixed in a beaker at 0.5% (w/v) in distilled water. The hollow spongy scaffolds were dipped into this sodium alginate solution for 30 minutes, and subsequently blotted against clean paper towels, removing the excess polymer from the scaffold. The scaffolds were cross-linked by dipping into 0.02M calcium chloride solution for 15 minutes. This cationic calcium ion then replaced the sodium ion, and thus formed insoluble calcium alginate over the hollow spongy scaffold [17]. These scaffolds were frozen at -20°C for 12 hours and then freeze-dried for 48 hours. After freeze drying, the scaffolds were stored at -20°C for chitosan loading at the center of the hollow spongy scaffolds.

The positive potential polymer solution: purified chitosan was mixed in a separate beaker at 1% (w/v) in 0.5% acetic acid solution until the complete dissolution of chitosan, to which 0.01% (w/w) genipin was added and allowed to stir for 48 hours at 37°C. The resulting solution was cross-linked in this process, followed by gelation. At the starting point of gelation, BCP powder at 1% (w/v) was added to the solution and allowed to stir overnight. This process prepared BCP loaded genipin crosslinked gelled chitosan solution. This gelled chitosan was loaded slowly at the center of the hollow spongy scaffold using a 10ml syringe, preventing unexpected flow around the shell of the spongy scaffold. The scaffolds were frozen at -20°C for 12 hours, freeze-dried for 48 hours, and stored at -20°C for BMP-2 loading and further investigation. Thus, three types of cylindrical scaffolds were synthesized: hollow BCP, BCP-0.5% Alg-1% CHT-BCP (BCP-PEL Hy), and BCP-0.5% Alg-1% CHT-BCP-BMP 2 (BCP-PEL Hy-BMP 2). Here PEL Hy is representing poly electrolytic hydrogel (0.5% Alg-1% CHT-BCP).

### 2.4 BMP-2 loading and release study

BMP-2 was loaded into the BCP-chitosan matrix located at the center of the fabricated scaffold. In brief, BMP-2 was mixed with 1% (w/v) bovine serum albumin solution with distilled water. After mixing, the total solution volume was 1ml. A total of 100μl was loaded 4 times into the BCP-chitosan matrix. BMP-2 was favored both for its adsorption within the BCP spongy scaffold and absorption within the cationic polymer, chitosan, at the center [11, 18].

This experiment applied a bipotential polymeric surface as a carrier of the bone-inducing protein BMP-2. BSA was used as a carrier protein. BMP-2 adsorption on the bipotential polymeric surface was studied by BSA loading efficiency. Two different concentrations of BMP-2, 0.1μg and 0.05μg were loaded into the positive chitosan core of the hollow spongy scaffold. BMP-2-loaded bipotential hollow spongy scaffolds were taken in triplicate and dipped into minimum essential media (MEM) inside a 37°C incubator. The extract solutions were collected at different time intervals as indicated in our results. The respective data were analyzed using a BMP-2 release kit (Quantikine ® Elisa), thus concentration of BMP-2 in the respective samples were determined by ELISA. Cumulative release was measured and expressed as a percentage of initial loading quantity.

### 2.5 Surface morphology analysis

Surface morphology of the samples was studied using SEM (JEOL, JSM-6701F, Japan). Source samples were prepared as stated above. Samples were prepared with appropriate thickness and sputter-coated with a platinum source before analysis. The study of chemical composition of the surface was performed by energy dispersive spectroscopy (EDS, Oxford instruments, UK) during SEM observation.

### 2.6 Chemical functional group analysis and scaffold parameters

Presence of calcium alginate at the shell and chitosan-BCP particles at the center of the spongy scaffold were studied in triplicate using Fourier transform infrared (FTIR) spectrometry (Spectrum GX,Perkin Elmer, USA). We had followed standard machine settings. Briefly, the infrared spectra of the samples were investigated over a wavelength range of 3000-500cm^-1^, and all the spectra were taken in the spectral range by the accumulation of 64 scans with a resolution of 4cm^-1^. The phase analysis of the BCP powder and the sintered scaffold were studied using X-ray diffraction (XRD, D/MAZ-250, Rigaku, Japan). XRD patterns of the samples were carried out at 35kV and 15mA, with a Cu Ka radiation wavelength of λ = 1.5406 A˚. Diffractograms were obtained from 20 to 65 on a 2Φ scale with a step size of 0.02. The phase ratio was determined using XRD analysis software PDXL (together with phase identification) and employing the Reference Intensity Ratio (RIR) method. The phase ratio for multiple observations was found to be HAp: TCP 60–70: 40–30 [6].

We have used a mercury porosity meter (PoreMaster TM, Quantachrome Instruments, FL, and USA) to investigate the porosity and average pore size. These investigations were performed in triplicate. The compressive strength was measured from the dry cylindrical spongy scaffolds (Diameter; 4mm, length; 12mm) in triplicate. We have used a universal testing machine (UTM, R&B UNITECH-T, Korea) with a crosshead speed of 0.5 mm/min under ambient conditions. This machine used Helio-X software to finally determine the compressive strength (a mean value from three trials) and expressed as the mean ± standard deviation (SD).

### 2.7 Degradation behavior of the polymer matrix

Hollow spongy scaffolds with a diameter of 3mm were prepared in triplicate as described in the former section, coated with calcium alginate at the shell, and loaded with genipin crosslinked chitosan at the center. Degradation behavior of this complex was analyzed by dipping the scaffolds into PBS (pH 7.4) in a 37°C incubator for 1, 3,7,13,21 and 28 days. We have taken 1ml PBS solution containing 1.5 μg/ml lysozyme (hen egg-white, Sigma-Aldrich, Oakville, Canada). The concentration of lysozyme was chosen as the concentration in human serum. The lysozyme solution was refreshed daily to mimic the body function. This study was started with measuring dry weight (W_0_). And after incubation for respective days, sample weight (W_t_) was taken at dry condition. Percentage of loss weight was calculated as (W_0_-W_t_)/W_0_ in percentage. The respective data were plotted as a curve using the mean ± SD of the population. In addition to the weight loss ratio, we had taken SEM image to confirm the degradation of bipotential polymer.

### 2.8 *In vitro* study

#### MC3T3-E1 pre-osteoblast cell culture

Pre-osteoblast MC3T3-E1 cells, derived from mouse, were obtained from the American Type Culture Collection (ATCC CRL-2593), and cultured in α-MEM (HyClone, Logan, UT) supplemented with 10% fetal bovine serum (FBS, Grand Island, NY) and 1% penicillin/streptomycin (antibiotics, Bio-Whittaker). MC3T3-E1 cells were maintained in a humidified incubator at 37^°^C and 5% CO_2_ (incubator, ASTEC, Japan).

#### Cell viability using an MTT assay and a Live/Dead Cell Kit

Approximately 10^4^ cells/ml were seeded on the scaffolds in 24-well plates. Three types of samples were taken in triplicate for MTT and Live/Dead study. We have used 1ml solution for each well. All wells were seeded with cells in static condition. The plates were incubated in a CO_2_ incubator for 1, 7 and 14 days. At the end of the respective days, 100μL 3-[4, 5-dimethylthiazol-2-yl]-2, 5-diphenyltetrazolium bromide (MTT, Sigma, 5mg/ml in phosphate buffered saline) solution were added to the wells and incubated for 4 hours. Afterwards, the settled formazan was solubilized in 100μl DMSO, the O.D. values of the solution were measured using an ELISA reader (EL, 312, Biokinetics reader, Bio-Tek instruments) at a wavelength of 595nm. It corresponds to the number of viable cells. By this way, cell proliferation on different days was quantified. The ratio of live/dead cells (LIVE/DEAD Kit: 0.1mM Calcein AM, 0.4mM ethidium bromide homodimer-1, Invitrogen) was observed after 7 days of culture.

#### Cell morphology analyses

For the study of cell morphology, preosteoblast MC3T3-E1cells (10^5^ cells/ml) seeded scaffolds (static Condition) were labeled with fluorescence conjugated antibody. All the samples were taken in triplicate for each category. In short, after 7 days of culture (in a humidified incubator provisioned with 5% CO_2_, 37ºC), the scaffolds were rinsed twice with PBS followed by fixation in 4% paraformaldehyde (Sigma-Aldrich) for 15 minutes at room temperature. Subsequently, the cells were permeabilized with 0.25% Triton X-100 (Sigma-Aldrich) for 10 minutes, and blocked with 1% BSA for 1 hour. Cytoskeleton of the cells were stained using a Fluorescein isothiocyanate (FITC) conjugated Phalloidin (25μg/ml-Sigma) for 2 hours at room temperature and nuclei were counterstained with 1μg/ml HOECHST 33342 (Sigma). The scaffolds were visualized using a confocal fluorescence microscope (Olympus, FV10i-W). Finally, images were analyzed using the accompanying software (FV10i-ASW 3.0 Viewer).

#### Quantitative real-time PCR

Total RNA was isolated using Trizol Reagent (life technologies, Invitrogen, Carlsbad, CA, USA) and the RNA concentration was calculated with the absorbance at 260nm and 280nm using a Nanophotometer (IMPLEN, Germany). For this study, 9 well cell culture plates were taken. In each well, three samples of each category were seeded with preosteoblast MC3T3-E1cells (10^6^ cells/ml) for 7 and 14 days. Three categories were arranged in triplicate. We had used cell culture plate as a control. To obtain cDNA, equal amounts of total RNA were reverse transcribed using the iScript^TM^cDNA Synthtesis Kit (Bio-Rad, USA). Real-time PCR was conducted using the Bio-Rad Real Time PCR System (Bio-Rad Laboratories, Hercules, CA, USA) in a 20μl reaction volume containing 10μl SYBR Green ER^TM^(Invitrogen, Carlsbad, CA, USA), 10pmol forward primer, 10pmol reverse primer, and 1μg cDNA. The primers used for the detection of the genes were designed for alkaline phosphatase (ALP), osteopontin (OPN); osteocalcin (OCN), collagen type I (COL I) and glyceraldehyde 3-phosphate dehydrogenase (GAPDH) and given in Table 1. The mouse GAPDH gene was used as an internal control. Real-time PCR was performed as follows: 40 cycles of denaturation at 95°C for 30 seconds, annealing at 58°C for 30 seconds, and extension at 72°C for 30 seconds. The fluorescence resulting from the incorporation of SYBR Green 1 dye into the double-stranded DNA during the PCR, and the emission data, were quantified using the threshold cycle (C_t_) value. Data were normalized to *GAPDH* and presented as the mean fold change compared with control.

**Table 1:** Primer sequences used for the detection of the genes.

Gene	Primers
GAPDH	F: 5′-TCTCCTGCGACTTCAACA-3
	R: 5′-CTGTAGCCGTATTCATTGTC-3′
Alkaline phosphate (ALP)	F: 5'-CCAGCAGGTTTCTCTCTTGG-3′
	R: 5′-GGAATGTTCCATGGAGGTTG-3′
Osteopontin (OPN)	F: 5′-GATGATGATGACGATGGAGA-3′
	R: 5′-GACTGTAGGGACGATTGGA-3′
Osteocalcin (OCN)	F: 5'-GCTTAACCCTGCTTGTGA-3′
	R: 5′-TCCTAAATAGTGATACCGTA-3′
Collagen Type I (COL I)	F: 5’-GTGAGACAGGCGAACAAG-3’
	R: 5’-CAGGAGAACCAGGAGGAC-3’

#### Immunoblotting

MC3T3-E1 cells (10^6^ cells/ml) were cultured on scaffolds for 7 and 14 days. Samples were arranged in a similar fashion as described in quantitative real-time PCR. The cells were harvested using a lysis buffer (RIPA, Sigma-Aldrich), vortexed, and centrifuged for 10 min at 13000 rpm and 4°C. Aliquots (20 μg) of the proteins were analyzed via western blotting using a 1:100 dilution of anti-collagen type I (COL I) antibody, alkaline phosphate (ALP) antibody and osteopontin (OPN) antibody (Santa Cruz). An anti-β actin antibody (1:500) was used as the loading control. The samples were subjected to SDS-PAGE on 12% gels, which were then transferred to polyvinylidene difluoride membranes (Millipore). The membranes were blocked with 5% fat-free milk at room temperature before incubation with optimal dilutions of primary antibody overnight at 4°C. The immunocomplexes were visualized using the enhanced chemiluminescence reagent (Amersham) in accordance with the manufacturer’s instructions

### 2.9 *In vivo* study

#### Rabbit radius surgery

The rabbit model study for invivo investigation of the fabricated long bone scaffold was approved by the “Institutional Animal Care and Use Committee (IACUC)” of Sonchunhyang University. All implantations in the animals were performed with the approved guidelines of the Soonchunhyang University IACUC.

A total of 12 New Zealand white rabbits (3kg each) were purchased and kept with normal diets and movement. Surgery was done after one week of acclimatization and all of them survived until the scheduled date of sacrifice. General anesthesia was performed on before the surgery using an intramuscular injection of a mixture of Zoletil 50 (Virbac, Mexico). Limbs were shaved, skin was disinfected with iodine solution, an incision was made to expose the radius, and a 12 mm segmental bone defect was created with a trephine drill [19, 20]. The three types of cylindrical scaffolds: hollow BCP, BCP-PEL Hy, BCP-PEL Hy-BMP 2 were then press-fitted into the radial defects. Two samples under each category (a total of 12 samples) were implanted for 4 and 8 weeks. We had avoided the control (a large segmental defect model without any sample) due to the inherent instability of the defect without any implant and due to a potential insignificant healing that would happen from it. A PCL-Gelatin electrospun mat was inserted around the defect site to inhibit the bone cells from surrounding the host bone. The surgical site was finally covered with an adhesive bandage. 0.2 ml antibiotic was intramuscularly administered to each rabbit on the first postoperative day, in order to prevent infection. At a predetermined time point, the animals were anaesthetized and sacrificed. After the respective time periods, the extracted samples were preserved in 10% buffered formaldehyde solution and later analyzed by micro CT, followed by a decalcification process with a 5% nitric acid solution for H&E and Masson’s trichrome staining.

#### Micro CT

Micro CT data were captured under a standard setting. Briefly, high-resolution micro-CT used multiple angular views that helped to quantify X-ray absorption within each cubic voxel element of the scanned volume. Image data were acquired, reconstructed, and analyzed using the Skyscan software (Skyscan 1076, Antwerp, Belgium). It could present data as 2D axial, sagittal, and coronal cross-sectional images. The version of Skyscan in application had a Windows-based graphical interface for image reconstruction, data visualization, and quantitative analysis [21–23]. To investigate bone regeneration inside the sample, a circular region of interest (ROI) covering the implantation site was chosen for quantitative analysis. A total of 570 slices were used for CT analysis. To evaluate bone healing efficacy, Micro CT was performed at 4 and 8 weeks. We have fixed voxel size to be 10 μm. To define the newly formed bone, we had set our pixel range that closely matched with implanted scaffold and secondly, the native bone. From the reconstructed 3D image based on this pixel range, the bone formation was shown.

#### H&E and Masson’s trichrome staining

5 μm sections were placed over a glass slide and routinely stained with hematoxylin & eosin (H&E) coupled with Masson’s trichrome stain. In short, H&E staining involved several stages such as: washing with xylene followed by hydration of the sample slides, dipping into hematoxylin solution for 10 minutes, washing and dipping into 1% hydrochloric acid (HCl) & ammonia NH_3_ solutions, dipping into an eosin solution for 3 minutes, and finally dehydration and washing in xylene. And Masson’s trichrome staining involved washing in running tap water followed by staining with Weigert’s iron hematoxylin working solution for 10 minutes and then washed in warm tap water for a further 10 minutes. Afterwards, samples were stained in Biebrich scarlet-acid fuchsin solution for 15 minutes and then in distilled water. Subsequently, samples were differentiated in a phosphomolybdic-phosphotungstic acid solution for 10–15 minutes, stained with aniline blue for 3 minutes and further differentiated in a 1% acetic acid solution for 2 minutes [24] and finally dehydration and washing in xylene.

### 2.10 Statistical analysis

All statistical analyses were performed using GraphPad Prism version 5.00 for windows. Differences among groups were assessed by two way ANOVA analysis. The analysis was followed by Bonferroni post-hoc test. Results were expressed as mean ± standard deviation. The significance levels were designated as (*, P<0.05; ** P<0.01; *** P<0.001).

## Results

### 3.1 Structure and Surface Morphology

**Fig 1:**
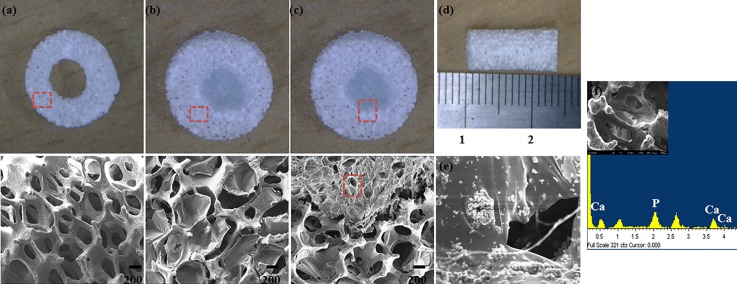
Surface Morphology (a) Spongy Scaffold (BCP) (b) 0.5% Alg-1% CHT-BCP (BCP-PEL Hy) (c) 0.5% Alg-1% CHT-BCP-BMP-2 (BCP-PEL Hy- BMP 2) (d) 12mm Scaffold for In Vivo (e) BCP loaded Chitosan at core (f) EDS profile for spongy scaffold.

The hollow spongy scaffolds were coated with 0.5% (w/v) sodium alginate cross-linked by calcium chloride, and subsequently loaded with 1% (w/v) BCP-Chitosan polymer complex (cross-linked by genipin). The samples: hollow BCP, BCP-PEL Hy and BCP-PEL Hy-BMP-2 were observed by SEM. EDX analysis was performed to identify the sample composition. These data are shown in Fig 1. To determine the pore size we took several SEM images (same magnification) of individual samples, manually measured the size of the pores in each image, and took the average of multiple readings. To ensure we encountered the median, the diameters of the pore geometry were taken from visual observation. From the SEM images, the hollow BCP spongy scaffold had an average pore size (214 ± 54μm) with the highest interconnectivity. This template scaffold was modified by coating with calcium alginate at the shell to give a negative potential, after which the average pore size changed to (148 ± 44μm). This template was further modified by the loading of Chitosan-BCP at the core, with an average pore size of 67.5 ± 22.33μm. These data are shown in Fig 1.

### 3.2 Chemical functional group analysis and scaffold properties

**Fig 2:**
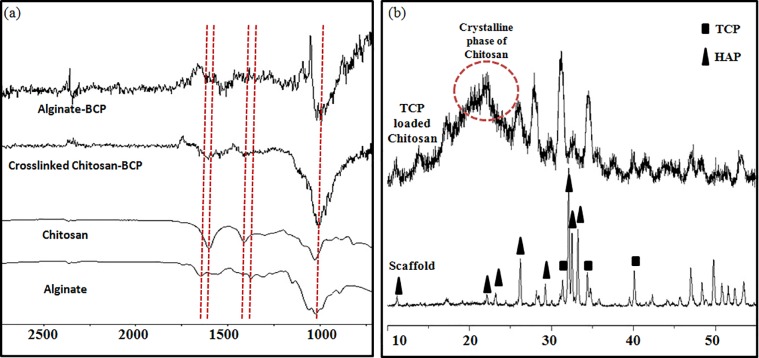
FTIR transmittance and XRD of the fabricated scaffolds.

**Fig 3:**
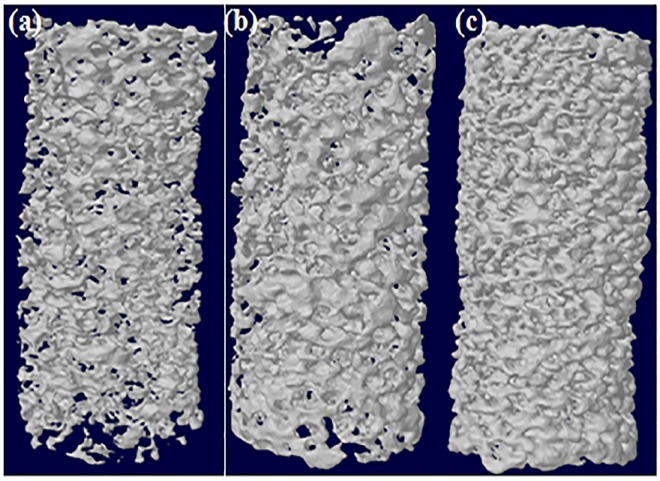
3D model for pore size orientation (a) Spongy Scaffold (BCP) (b) 0.5% Alg-1% CHT-BCP (BCP-PEL Hy) (c) 0.5% Alg-1% CHT-BCP-BMP-2 (BCP-PEL Hy-BMP2).

Bipotential polymer-loaded BCP spongy scaffolds were observed by Fourier transform infrared spectroscopy (FTIR) and XRD analysis. In hydrogel composed of chitosan, calcium alginate, BCP powder, several functional groups would be expected: -NH_2,_ C = O, O-H, and C-O-H. The FTIR spectrum is presented in Fig 2(A). Chitosan was revealed by the appearance of peaks at 1550cm^-1^ for -NH_2_ stretching, and calcium alginate was revealed by the appearance of peaks at 1415cm^-1^ for COO- stretching, and 1035cm^-1^ for C-O stretching. BCP is revealed by the appearance of a peak at 1035cm^1^ [25]. The diffraction pattern presented in Fig 2(B) confirms the presence of biphasic calcium phosphate (BCP) inside the sample. Since FTIR and X-Ray diffraction confirmed the respective functional groups inside the bipotential polymer-loaded spongy scaffold, various physical properties such as porosity (%), average pore size (μm), and compressive strength in dry conditions (MPa) were calculated for further characterization of the polymer-loaded spongy scaffold (Table 2 and constructed 3D image in Fig 3). From the data, a decreasing trend in porosity (%) and average pore size was evident with respect to the functionalization of the polymer scaffold. An increase in compressive strength was evident. The higher value of compressive strength for bipotential polymer-loaded scaffolds resulted from the matrix formation between the polymer and the ceramic scaffold, causing uniform distribution of stress around the scaffold.

**Table 2:** Physical characterization.

Sample	Scaffold Porosity (%)	Average pore size (μm)	Compressive Strength in dry condition (MPa)
Hollow BCP	94.18 ± 0.02	214 ± 54	0.2605 ± 0.10
0.5% Alg-BCP	82.22 ± 0.01	148 ± 44	1.13 ± 0.04
0.5% Alg-1% CHT-BCP	78.19 ± 0.03	67.5 ± 22.33	1.23 ± 0.03

### 3.3 Degradation behavior

**Fig 4:**
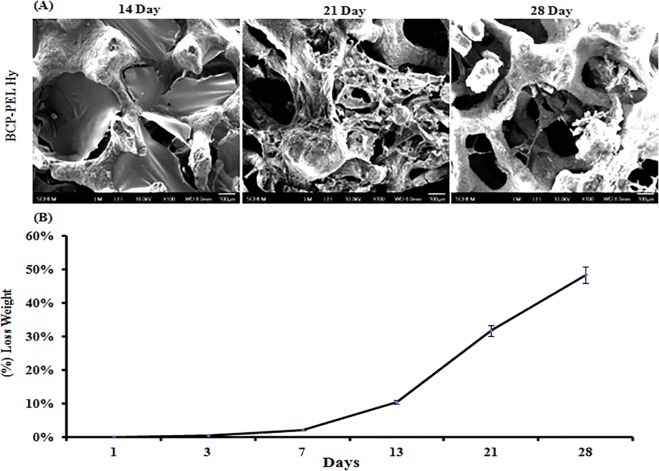
(A) Lysozyme activated degradation Behavior of BCP-PEL Hydrogel system. (B) Degradation behavior.

Dissociation of the scaffolds was observed through the degradation behavior of the polymer around the spongy scaffolds, which was measured by weighing the scaffolds after dipping into PBS solution on the indicated days. The degradation plot in Fig 4A and 4B shows that the BCP-PEL Hy samples still had 49% of their weight remaining after 28 days, indicating prolonged stability of the calcium alginate at the shell and of the genipin cross-linked chitosan-BCP at the core.

### 3.4 Protein release behavior

**Fig 5:**
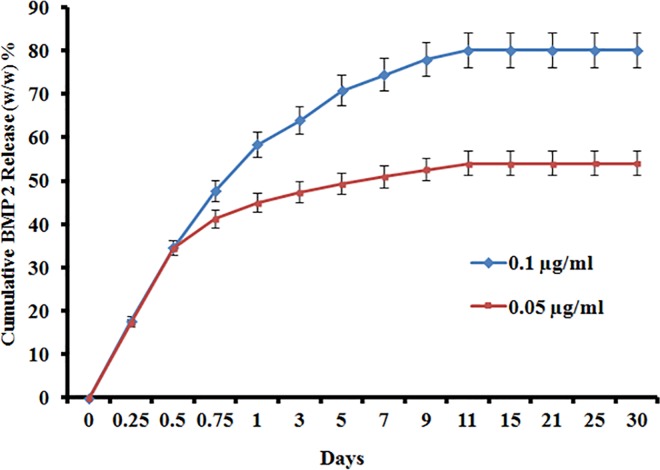
Cumulative BMP-2 release from BCP-PEL HY-BMP 2 system.

The *in vitro* release kinetics of BMP-2 from the scaffolds was interpreted by the cumulative amount and percentage of BMP-2 as a function of time. Fig 5 shows the cumulative release of BMP-2 from the BCP-PEL Hy-BMP 2 scaffold. The release velocity of BMP-2 increased at higher concentrations. The data shows a higher percentage of BMP-2 release occurred with 0.1μg BMP-2 compared with 0.05μg. An initial burst of release happened upto 5 days. The data represented a sustained release over a 10 days period. Our data plot covered a 30 days period. For 0.1μg BMP-2, the data plot showed that the release percentage of BMP-2 was 17.22% (w/w) after 6 hours and increased significantly to 78.48% (w/w) after 7 days. After a 15 day period, it reached to 80% (w/w) of the total BMP-2 content. However, over the remaining time, the release was insignificant. For lower concentration of BMP-2, we have found difficulty to detect the BMP-2 that remains into the sample. Strong immobilization of BMP-2 into the polyelectrolyte matrix (Chitosan-Alginate) is might be the reason for this.

### 3.5 *In vitro* study

#### Proliferation of MC3T3-E1 cells on scaffolds

**Fig 6:**
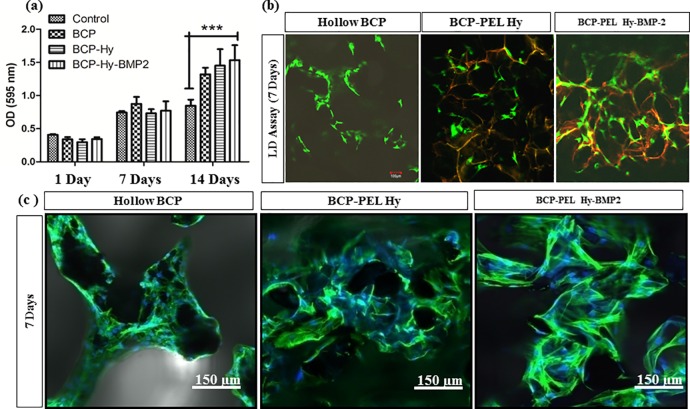
Cell Viability and Proliferation of spongy Scaffold: (a) MTT Assay (*, P<0.05; ** P<0.01; *** P<0.001) (b) Live dead Assay (c) F-actin Assay.

The cell viability and proliferation of the spongy scaffolds were characterized after 1, 7 and 14 days of culture. As seen in Fig 6A, the MTT assay for the hollow BCP, BCP-PEL Hy and BCP-PEL Hy-BMP 2 samples showed: after 1 and 7days, the OD values of respective samples were not statistically comparable, however after 14 days, a significant increase (p˂0.001) in OD values compared with the tissue culture plate was revealed that indicated a favorable biocompatibility.

The live/dead assay was performed after 7 days of culture. As seen in Fig 6B, an increasing trend of live cells was exhibited for the hollow BCP, BCP-PEL Hy, BCP-PEL Hy-BMP 2 samples, confirming a favorable interaction between the cells and the bipotential bioactive surface, resulting in faster cellular functionality. The increasing trend was due to the presence of optimum pore size distribution for effective initial attachment around the scaffolds. The higher porosity percentage resulted in a significant level of internal cellular migration.

The F-actin assay revealed distinct morphological and biochemical interactions with the scaffold, as shown in Fig 6C. The actin filaments were stained green to reveal the association with cells. The F-actin assay showed cellular spreading for the hollow BCP, BCP-PEL Hy and BCP-PEL Hy-BMP-2, however BCP-PEL Hy and BCP-PEL Hy-BMP-2 showed a significantly higher cell proliferation compared with the hollow BCP sample. Hence, BMP-2 signaling has a strong positive effect on cellular growth. It was indicated as higher cytoskeletal expression from F-actin assay.

#### Differentiation of MC3T3-E1 cells on scaffolds

**Fig 7:**
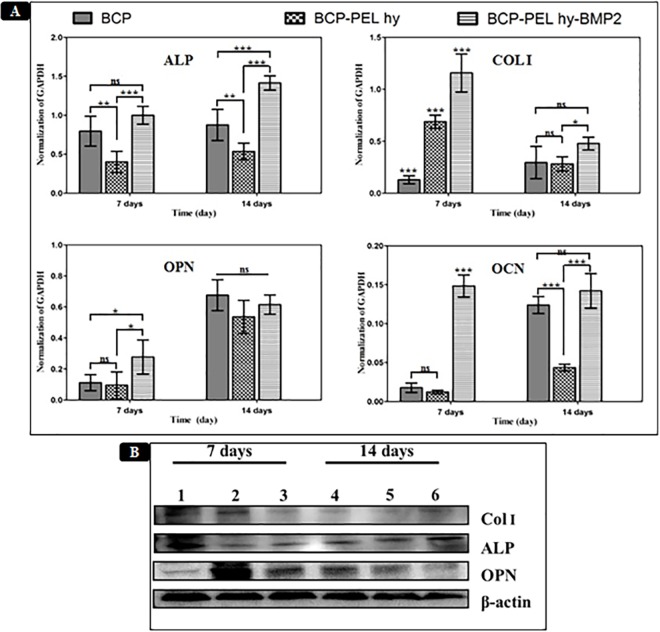
(A) The expression of osteopontin (OPN); alkaline phosphate (ALP); osteocalcin (OCN) and collagen type 1 (COL I) gene of MC3T3-E1cells for BCP; BCP-PEL hy and BCP-PEL hy-BMP 2 after 7 days and 14 days of cell culture in osteogenesis media, observed by real-time PCR. (*p< 0.05; **p<0.01; ***p<0.001; ns: no significant different). (B) The expression of osteopontin (OPN); alkaline phosphate (ALP) and collagen type 1 (COL I) protein of MC3T3-E1cells for BCP (1, 4); BCP-PEL hy (2, 5) and BCP-PEL hy-BMP 2 (3, 6) after 7 days and 14 days of cell culture in osteogenesis media, observed by Western Blot. β-actin levels are used as internal control.

The bioactivity of the scaffolds were determined by investigating the induced alkaline phosphatase (ALP), collagen I (COL I), osteopontin (OPN) and osteocalcin (OCN) activity of MC3T3-E1 cells. As demonstrated in Fig 7A, after 7 days of culture the ALP activity of MC3T3-E1 was significantly higher for the hollow BCP (p**<**0.01) and BCP-PEL Hy-BMP 2 (p**<**0.001) scaffolds compared with the BCP-PEL Hy scaffold. However, after 14 days of culture, the ALP expression continued to increase significantly for BCP-PEL Hy-BMP 2 scaffold (p**<**0.001) compared with the hollow BCP and BCP-PEL Hy scaffolds. This indicated the strong positive effect towards bone formation resulted from BMP 2 signaling. COL I was significantly expressed in all the three scaffolds specially in BCP-PEL Hy-BMP 2 scaffold (p**<**0.001) after 7 days of culture. However, after 14 days, COL I had an increased expression, nevertheless it was not significant compared with the hollow BCP scaffold. OPN expression was significantly higher in the BMP-2-loaded scaffold (p**<**0.05) compared with the other two samples after 7 days of culture. After 14 days, all the samples had increased expression. OCN was significantly expressed in the BCP-PEL Hy-BMP 2 scaffold (p**<**0.001) after 7 days of culture, which continued to increase after 14 days as compared with the BCP-PEL Hy scaffold.

#### Protein expression

The protein expression of collagen I (Col I), alkaline Phosphatase (ALP), Osteopontin (OPN) are presented in Fig 7B. Data show early expression of collagen I in the hollow BCP and BCP-PEL hy scaffolds. Alkaline phosphatase (ALP) was expressed early in all samples. Osteopontin (OPN) expression was significantly higher in the BCP-PEL Hy and BCP-PEL Hy-BMP 2 scaffolds.

### 3.6 Micro CT

**Fig 8:**
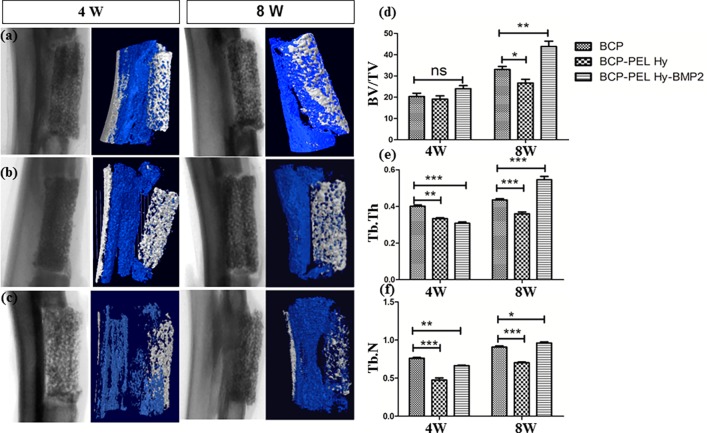
3D Model for new bone in growth from CT analysis for 4W and 8 W: Radiographic image and bone in growth of (a) BCP-PEL Hy-BMP 2 (b) BCP-PEL Hy (c) BCP; Quantitative data: (d) Bone volume/Tissue volume ratio (BV/TV) (e) Trabecular thickness (Tb. Th) (f) Trabecular number (Tb.N). (*p< 0.05; **p<0.01; ***p<0.001; ns: no significant different).

Qualitative micro-CT analysis showed no radiographic signs of inflammation such as diffusely delimited soft tissue infiltration, osteolysis, or osteomyelitis. At 4 weeks, 3D constructed CT images showed initial bone formation within the defect site for the hollow BCP and BCP-PEL Hy-BMP 2 samples. Minor bone formation was seen for the BCP-PEL Hy scaffold. At 8 weeks, the level of full defect bridging was significantly higher in the hollow BCP (p**<**0.05) and BCP-PEL Hy-BMP 2 (p<0.01) scaffolds compared with the BCP-PEL Hy scaffold. Here defect bridging indicated an intermediate interphase for the migration of host cells towards implantation site. Bone volume density is an important parameter to assess the comparative outcomes. The morphometric parameters using CT analysis are shown in Fig 8. An easy fragility and degradation were revealed for hollow BCP spongy scaffold. Bone volume density (bone volume/tissue volume, BV/TV), trabecular thickness (Tb.Th) and trabecular number (Tb.N) followed an increasing trend. Here bone volume/tissue volume (BV/TV) indicated new bone formation and cellular penetration, trabecular thickness (Tb.Th) presented bone micrograph and bone strength and trabecular number (Tb.N) presented porous structure. After 4W, trabecular thickness (Tb.Th) was significantly higher in Hollow BCP scaffold: from BCP-PEL Hy (p<0.01) and from BCP-PEL Hy-BMP2 (p<0.001). However, after 8W, trabecular thickness exhibited significantly higher in BCP-PEL Hy-BMP2 scaffold ((p<0.001) compared with Hollow BCP. Similar effect was exhibited for trabecular number (Tb.N). After 8W, it was significantly higher in BCP-PEL Hy-BMP2 scaffold ((p<0.05) compared with Hollow BCP.

### 3.7 Histological evaluation

**Fig 9:**
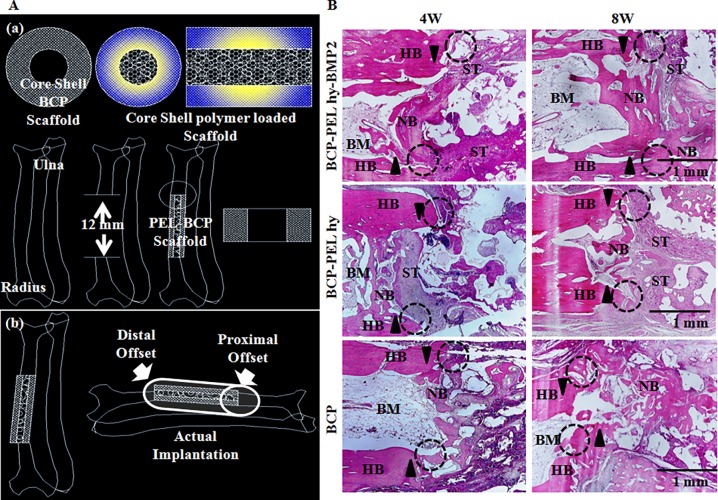
(A)(a) Schematic diagram of implantation (b) Actual implantation Site; (B): New bone in growth by H&E staining for 4W and 8W. Here HB, NB, BM and ST stands for host bone, new bone, bone marrow and soft tissue. Encircled region is enlarged in Figs 10 and 11.

**Fig 10:**
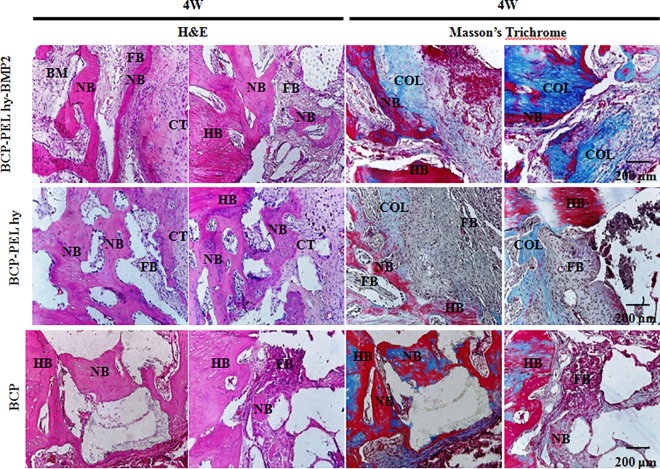
Cellular activity at scaffold-host bone interphase at 4 W time period. Here HB, NB, BM, CT, FB and COL stands for host bone, new bone, bone marrow, cartilage template and collagen.

**Fig 11:**
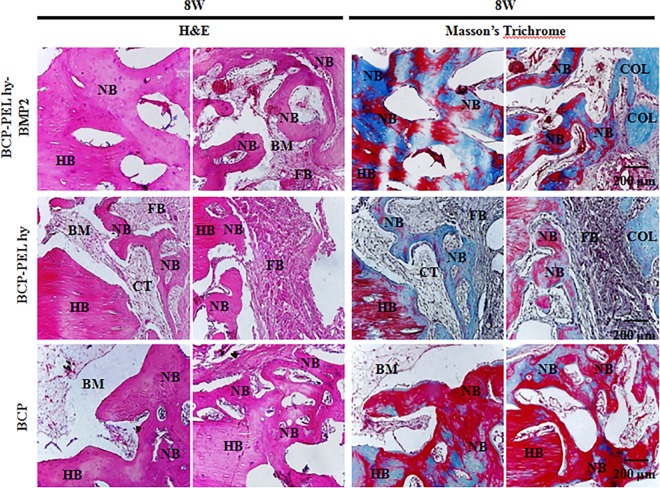
Cellular activity at scaffold-host bone interphase at 8 W time period. Here HB, NB, BM, CT, FB and COL stands for host bone, new bone, bone marrow, cartilage template and collagen.

Three types of scaffolds were implanted at radial section. Implantations were performed without any fixation plate. Our sample fabrication process limits to mimic native bone structure in proper surface topography and operational complexity prevents in mimicking average radial dimension. Due to the limitation of customizable scope, the scaffolds are not perfectly aligned at proximal and distal ends. This is shown in Fig 9A. After 4 weeks of implantation, the three types of scaffolds: hollow BCP, BCP-PEL Hy and BCP-PEL Hy-BMP 2 were surrounded by collagen fibers commonly referred to as connective tissue. Initial poor vascularization inside the defect site introduced the generation of intermediate cartilage instead of direct osteoblastic differentiation [26], resulting in chondrogenesis, and the migration of chondrocytes into the collagen capsule, which lead to the formation of woven bone. At 4 weeks, the formation of woven bone was confirmed by H&E and Masson’s trichrome staining. This data is presented in Figs 9B and 10. The rate of formation of woven bone was faster in the BCP spongy scaffold due to the favorable immunogenic response at the beginning. This newly formed woven bone in turn leads to the start of vascularization, inducing bone ossification through the coupling of chondrogenesis and osteogenesis via the endochondral pathway [27, 28]. But fibroblast cells penetration inside the scaffolds were lower compared with BCP-PEL hy-BMP 2. At 8 weeks, guided bone regeneration and significant bioresorption had occurred in all three samples, in addition, surrounding collagen fibers were still in place for the BCP-PEL Hy and BCP-PEL Hy-BMP 2 samples. Increased vascularization occurred in the BMP-2-loadedand BCP spongy samples. As a result, the formation of trabecular bone volume was significantly increased, being confirmed by H&E and Masson’s trichrome staining highlighted in Figs 9B and 11. This trabecular invasion may affect cortical bone formation at increased time intervals.

## Discussion

The present work aims to investigate the possibility of a segmental bone defect healing behavior by a modified hollow BCP scaffold. The scaffold was modified by the coating of positively charged polymers at the core (chitosan) and negatively charged polymers (calcium alginate) at the shell. Thus, the total composition acted as a bipotential polymeric surface around the scaffold. The presence of positively charged functional groups (crosslinked chitosan) can induce hydrophobicity at the core. Moreover, BCP loading into chitosan matrix enabled additional hydrophilic tendency together with improvement of biocompatibility. This bifunctional activity, coupled with sustain release of BMP-2, is conducive for the bone marrow for better cellular adhesion, migration, differentiation and related gene expression at the shell for various kinds of bone marrow cells [29]. This osteoinductive environment induces guided bone healing for segmental bone defect. This biochemical agent triggers signaling pathways that affects the osteogenic differentiation of stem cells inside the bone marrow that occurs through the chondrogenic pathway [30, 31]. Irrespective of the locations or stages, cartilage participates in the form of growth cartilage within developing or growing bones.

In the present work, we investigate the bone regeneration efficacy of porous hollow scaffolds with pore size modification through bipotential polymer coating and its application for segmental bone defect healing. Fig 1 shows the surface morphology of the hollow BCP scaffold, and the shell and core of the BCP-PEL Hy scaffold. Coating with electrolytic polymers: Chitosan, alginate at the core and shell significantly reduced the pore size to 148 ± 44μm and 67.5 ± 22.33μm, respectively, and this polymer modification also significantly increased the compressive strength to 1.13 ± 0.04MPa, as shown in Table 2 and Fig 3. This significant reduction in pore size aided cell attachment around the scaffold, and can retain the bone marrow cells in order to induce effective vasculogenesis inside the implant. This osteoconductive synthetic bone graft becomes osteoinductive as a result of BMP-2. In the presence of osteogenic cells from the host body, the total composite induces active stimulation of bone regeneration [32]. Hence, total composite becomes an osteoconductive template that can effectively mimic the microstructure of large segmental bone defects.

The osteoinductive performance of the scaffold is related to the adsorption kinetics of BMP-2 into the positively charged BCP-loaded chitosan matrix at the core of the hollow scaffold. BMP-2 (isoelectric point: 8.5, molecular weight: 32kDa) is a positively charged protein [33]. The positively charged chitosan was cross-linked into a gel and loaded into the core of the hollow spongy scaffold. The thermal stability of the gelled chitosan was studied by lysozyme-triggered degradation in a 37°C incubator. From Fig 4A and 4B, it was revealed that the degradation of the polymer was 49% after 28 days of incubation. This stability of the bipotential polymer around the hollow spongy scaffold would sustain the release of BMP-2 that was loaded with BSA (isoelectric point: 4.7, negatively charged protein), hence electrolytic absorption occurred into the chitosan matrix. As the initial burst of release of BMP-2 has the ability to attract osteoprogenitor cells into the delivery system, it can function as a chemoattractive protein for the recruitment and condensation of osteoprogenitor cells into the scaffold [34]. This would be helpful for faster healing of large segmental bone defects. The cumulative BMP-2 release is represented in Fig 5, showing the initial burst of release in 3 days of 64.04%, followed by the sustained release.

Initial biocompatibility of the scaffolds was measured by an MTT and a live/dead assay to investigate cell proliferation as shown in Fig 6. The biocompatibility of the scaffolds was significantly higher compared with the control MC3T3-E1 cells. This created a favorable environment in the host body immediately upon implantation. However, cell migration around the scaffold warrants further investigation regarding the intrusion of the vascular network into the scaffold. This may be a function of gene expression as well as a vasculogenesis capability of the scaffold. Osteogenic gene expression such as: ALP, COL I, OPN and OCN in MC3T3-E1 cells was measured. The cell response to the growth factor BMP-2 was evaluated by the presence of early expression of COL I and OPN to a significant level. From the gene expression data presented in Fig 7A, COL I and OPN was expressed after 7 days of culture with the MC3T3-E1 cell line. ALP had increased expression after 7 days, and was significantly expressed after 14 days of culture. ALP activity is a potent marker of early osteoblastic differentiation. Thus, ALP activity is considered as an indicator of the cell response to BMP-2, and its significant expression after 14 days indicates sustained delivery of BMP-2 [34, 35]. Fig 7B shows that the relevant protein expression (ALP, COL I and OPN) in consistent with gene expression. The present study shows that the marker of osteoblastic differentiation for bone mineralization, OCN, was significantly expressed after the first 7 days, and increased after 14 days, indicating early bone mineralization.

The fabricated bipotential polymer-coated hollow spongy scaffolds with BMP-2 had the ability to induce the necessary signals for fast healing, and act as a functionalized bone graft substitute for cell and bone growth. The scaffolds can mimic the structure of cancellous bone with a pore size gradient, providing an open and connected porous framework for cellular migration, extracellular matrix production, new bone formation and the neo-vascularization from adjacent bone tissue. Upon implantation, *in vivo* osteogenesis and integration with surrounding bone tissue is exhibited in Figs 9B and 10. The cascade of cellular events is as follows: cellular proliferation, differentiation, and extracellular matrix synthesis resulted from the regulatory roles of growth factors, TGF-β and PDGF synthesized by osteoblast and chondrocytes throughout the healing process [36]. Initially the defect region was surrounded by soft fibrous tissue. In the inflammatory stage, a hematoma developed within the fracture site during first few hours and days. Inflammatory and fibroblast cells infiltrated the implanted scaffold, enhancing the ingrowth of vascular tissue. Progression of this vascular tissue enhanced the formation of the collagen matrix, as exhibited in Figs 9B and 11. This infiltration of fibroblast cells promoted the differentiation into myofibroblasts that produced large amounts of extracellular matrix (ECM) proteins, such as type I collagen and fibronectin, contributing to the replacement of the granulation tissue and peripheral fibrosis of the wound [37]. The collagen-rich matrix, coupled with ALP expression, enhanced the hypertrophic chondrogenic profile [38]. This cartilage profile located at the periphery of the implant could instruct the surrounding mesenchymal cells to differentiate into osteoblasts [26]. BMP-2 can induce the formation of bone and cartilage through osteoblast and chondrocyte differentiation [39]. Hence, the BCP-PEL Hy-BMP-2 sample had the ability of faster bone healing, as evident from the significant expression of ALP, OPN and COL I, presented in Fig 7A. This significant expression resulted from the initial burst of release of BMP-2, initiating the endochondral pathway, which directed osteoblast and chondrocyte invasion into the defect site for the enhancement of vasculogenesis. This vasculogenesis triggered the osteoblastic differentiation inside the large segmental implant, with this bone formation being demonstrated by the H&E and Masson’s trichrome staining in Figs 9B and 10. The penetration of bone cells inside the large segmental implant was also confirmed with the micro CT data exhibited in Fig 8. Based on the similarity of close contrast of the native bone, computed tomography (CT) analysis generated a 3D model to show the bone formation along the longitudinal cross section of the implant.

However, the implanted spongy scaffold coupled with the limited compressive strength shortened the sustenance of the microstructure under normal loading situations. Loading behavior is very significant for bone adaptation to bone cells. Mechanical signals *in situ* can induce appropriate changes in the bone architecture during skeletal growth and development. An adaptive response is initiated from a short duration of mechanical loading, and immature bone growth is more responsive to alterations of cyclic strains than mature bone [40, 41]. Thus, guided bone regeneration around the scaffold geometry may be delayed as a result of an incomplete structured fibrous network around the implanted scaffold. Initially, significant expression of OCN presumptively induced mineralization as seen in Fig 7A, therefore, OCN expression confirmed that early mineralization occurred in the BMP-2-loaded sample. Subsequently, due to the absence of dynamic loading, guided mineralization was inhibited as a result of the absence of a structured fibrous network. Consequently, the formation of cortical bone was delayed. Under the prolonged passage of time, the degree of mineral particle orientation may have occurred, thus, the alignment of collagen type I fibers along the long bone axis ensued.

Further extension of this work may include an attempt to increase the strength of the scaffold so that it can retain its shape under prolonged dynamic loading with optimum bioresorption. This would enhance the guided bone regeneration along the long bone axis. The porous scaffold may be further functionalized with several growth factors such as TGF-β and VEGF with sequential release. In addition, *in vivo* implantation should perfectly fit into the defect cavity, and the scaffold should perfectly mimic the exterior periphery for effective guided bone regeneration.

## Conclusion

In the present work, we have investigated the efficacy of bipotential polymer modification of porous hollow spongy scaffolds for the healing of large segmental bone defects. We found that the BMP-2-loaded BCP-PEL Hy-BMP 2 scaffold has the ability to express genes for osteoblastic differentiation earlier the other types of scaffolds. An initial burst of release of BMP-2 increased ALP expression, resulting in enhanced chondrogenesis. Consequently, bone formation occurred via chondrogenic pathway. After 8 weeks of implantation, significant bone growth (42% as per BV/TV ratio) occurred with the BMP-2-loaded scaffold.
